# Progress of research on the relationship between efferocytosis and tumor

**DOI:** 10.3389/fonc.2024.1361327

**Published:** 2024-04-09

**Authors:** Xuexin Yao, Ling Zhang, Siyi Sun, Aishuang Fu, Yanlei Ge

**Affiliations:** North China University of Science and Technology Affiliated Hospital, Tangshan, China

**Keywords:** tumor, efferocytosis, tumor-associated macrophages, macrophages, tumor microenvironment

## Abstract

Tumors are genetic changes that develop in an organism as a result of many internal and external causes. They affect the biological behavior of cells, cause them to grow independently, and give rise to new, perpetually proliferating organisms. Recent research has supported the critical function of tumor-associated macrophages in the development, progression, and metastasis of tumors through efferocytosis. Yet, there is still much to learn about the mechanisms behind their contribution to tumor pathological processes. As a result, it’s critical to actively investigate how cytosolic processes contribute to the growth of tumors and to create novel therapeutic approaches.

## 1 Introduction

A tumor, which frequently appears as a localized aberrant tissue mass in the body, is a new organism created by the improper multiplication of cells under the influence of different tumor-causing substances and major problems in the regulation of cell growth. Generally speaking, a tumor results from the “mutiny” of human cells if all infectious diseases are thought of as the invasion of foreign organisms on the human body. The phrase “efferocytosis,” also known as “programmed cell removal,” refers to the process by which macrophages remove programmed dead apoptotic cells. The term “efferocytosis” is derived from the Latin word “efferre.” This process can be understood as the burial of apoptotic cells. One kind of macrophage engaged in efferocytosis is called tumor-associated macrophages (TAMs) (1, 2). The immune-silencing clearance of apoptotic cells by efferocytosis has been shown in recent studies to play a significant role in the tumor microenvironment, tumor progression, and metastasis (3, 4). This process also promotes inflammatory catabolism and immunosuppression and gives cancer cells a safe haven from immune surveillance, which aids in the progression of tumors (5, 6). Therefore, one possible target for therapy and for regulating tumor progression and metastasis is to interfere with macrophage efferocytosis. In this study, we explore the mechanism of efferocytosis as well as the connection between the tumor microenvironment and efferocytosis.

## 2 The process of efferocytosis

A healthy body can remove billions of senescent, apoptotic, and necrotic cells every day, and cytokinesis is performed not only by specialized phagocytes, macrophages, and dendritic cells, but also by many non-specialized cells in the body, such as epithelial, endothelial, and fibroblasts. Even in tissues with a high cell turnover rate, dead cells do not accumulate, indicating a very efficient clearance, a process known as efferocytosis. This ability is a necessary process to maintain tissue homeostasis in normal physiology and to restore it in the event of disease (7, 8) In some chronic diseases, efferocytosis cells become defective and an accumulation of dead cells ensues (9) Apoptotic cells can become necrotic secondary to autoimmune deficiencies and pathological inflammation (10, 11).

**Figure 1:**
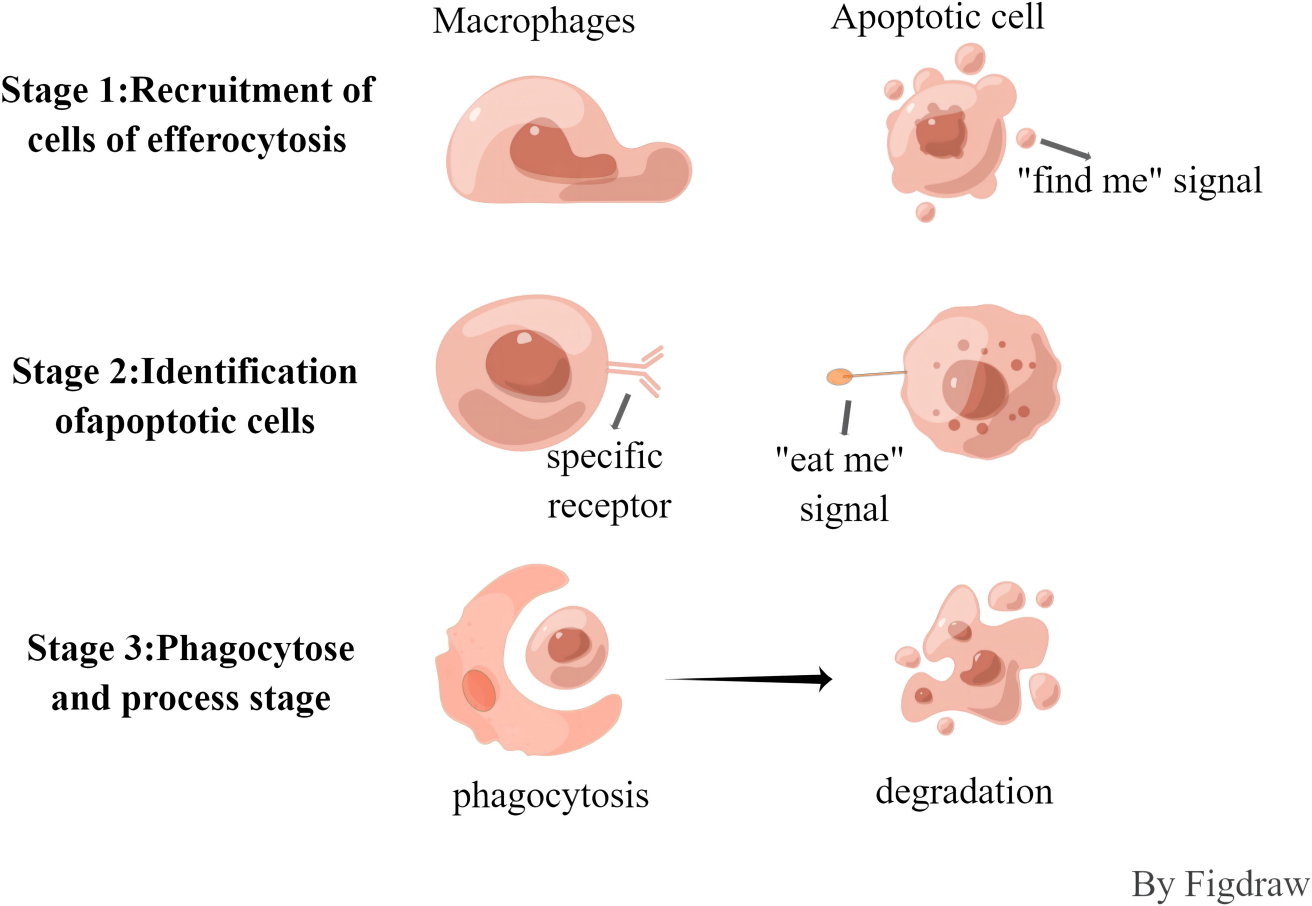
The process of efferocytosis.

The efferocytosis cells of apoptotic cells is synergistically regulated by a variety of signaling molecules, such as “find me, eat me and do not eat me”, and the process is roughly divided into three phases (**Figure 1**). In the first stage, the recruitment of buried cells, cells undergoing apoptosis will release the “find me” signal, which can be recognized by specific receptors on the surface of buried cells, and can induce the migration and aggregation of buried cells to the apoptotic cells. Currently known “find me” signaling molecules include hemolyzed lecithin(LysoPC), Sphingosine-1-phosphate (S1P), chemokine CX3CL1, and nucleotide ATP/UTP, and the surface receptors include the G protein-coupled receptor G2A family (G2A), sphingosine-1-phosphate receptor (S1PR), C-X3-C Motif Chemokine Receptor 1, and G protein-coupled receptor P2Y family (P2Y) (12, 13). In the second phase, the recognition phase of apoptotic cells, “eat me” signals are released during apoptosis that bind to receptors on the surface of buried cells. The most important “eat me” signal is phosphatidylserine (PS), which is exposed on the surface of apoptotic cells and binds directly or indirectly to receptors. Direct receptors include brain-specific angiogenesis inhibitory factor (BAI), T-cell immunoglobulin mucin receptor 4 (TIM4), scavenger receptor stabilin2, and myeloid-associated immunoglobulin receptor CD300f (14), etc., whereas the tyrosine kinase TYRO3/AXL/MERTK receptor binds to PS indirectly through some soluble bridging molecules. This phase is also negatively regulated by “do not eat me” signaling molecules, which have been identified as CD47, CD31, CD61, CD46, of which CD47 is the most important. and the living cells prevent themselves from being recognized by the buried cells through the interaction of CD47-SIRP1, and play the function of inhibiting the efferocytosis (13, 15, 16). In the third phase, phagocytosis and processing phase, the binding of apoptotic cells to receptors on efferocytosis cells triggers a complex cytoskeletal rearrangement that promotes the formation of phagolysosomes, which then fuse with lysosomes, which contain a number of pH-sensitive degrading enzymes, thus promoting the release and biodegradation of apoptotic cell contents. The processing of apoptotic material can be carried out either by the typical degradation program, whereby early phagosomes mature into late phagosomes that eventually fuse with lysosomes, or by microtubule-associated protein 1A/1B light chain 3 (LC3)-associated phagocytosis.

In contrast to apoptosis, non-apoptotic dead cells lose plasma membrane integrity and belong to a regulated form of necrosis, which is also activated by external apoptotic receptors (death receptors known to mediate exogenous apoptosis include members of the tumor necrosis family, including tNFr1, Fas receptor (CD95), and tumor necrosis factor-associated apoptosis-inducing ligand (traiL) receptor), and necrosis is initiated by the activation of riPK1, which upon phosphorylation binds and activates riPK3. riPK3-mediated phosphorylation of mixed-spectrum kinase structural domain-like proteins (MLKLs) promotes their oligomerization and insertion into the plasma membrane, forming a membrane-disrupting pore that leads to the release of death- and damage-associated molecular patterns (DAMPs) (17–19). DAMPs originate from cells and are released upon cell death, triggering an inflammatory response, and also act as chemotactic agents for macrophages, and they have multiple effects on macrophage action and immune activation.DAMPs are metabolically diverse entities that include genomic and mitochondrial DNA, nuclear proteins (high mobility group protein B, histones), cytoplasmic proteins (S100), cytokines (IL-1α, IL-33, IL-36) and other small molecules (ATP, UTP, uric acid crystals) (20, 21). The different modes of cell death of various cells have unique forms of activation and present different signals to phagocytes, followed by the initiation of associated clearance programs that lead to different formal physiological outcomes through efferocytosis.

## 3 Tumor microenvironment and macrophages

Tumor microenvironment (TME), which is crucial to the growth of tumors, is made up of blood vessels, fibroblasts, macrophages, B-cells, T-cells, and other host cells and components besides cancer cells (22, 23). It is now well established that macrophages make up a sizable fraction of the host leukocyte infiltration in most malignant tumors (24). TAMs are a flexible, varied population of cells in the TME that make up a sizable portion of some malignancies. TAMs influence the TME in a variety of ways that may impact tumorigenesis and development through the synthesis and release of a wide range of cytokines (25).Virchow originally saw leukocytes in 1863 (26), whereas Ellie Metchnikoff first recognized macrophages in 1882 (27). Bone marrow monocytes give rise to the majority of macrophages, an essential type of immune cells in the human body. As an essential part of innate immunity, it may directly phagocytose and eradicate invading pathogens such as bacteria, fungus, and parasites. Moreover, it possesses the capacity to discharge an array of immune constituents that can expose foreign antigens to T cells, elicit activation of supplementary adaptive immune cells, and facilitate the induction of adaptive immunity via antigen presentation (28).

Macrophage polarization, the transition between M1 and M2 types that happens when macrophages behave differently in distinct microenvironments, is controlled by a number of microenvironmental elements in addition to signals from tumor and stromal cells. The M1/M2 macrophage subtypes exhibit distinct functions, express corresponding genes, release distinct immunological markers, and cytokines (29). It is critical to human health that M1 and M2 macrophages coexist in equilibrium. But the appearance of illness could upset this equilibrium, causing an overreaction to inflammation or a reduced ability to heal wounds. For instance, in order to evade detection by the immune system, viruses have developed a number of clever strategies for causing a shift in the macrophage to an M2-skewed state during infections (30, 31). An imbalance in the polarization of M1/M2 macrophages in autoimmune disorders may worsen the condition and prolong chronic inflammation (32). Both M1-type and M2-type large cells are involved in atherosclerosis; M1 macrophages cause plaque rupture, while M2 macrophages aid in the calcification of the plaque (31, 33). M2-type macrophages primarily secrete anti-inflammatory cytokines like interleukin 10 (CXCL-10), interleukin 13 (IL-13), and interleukin 4 (IL-4), and express abundant arginine-1, mannose receptor (MR, CD206), and scavenger receptor, which contribute to tumor growth and spread. In contrast, M1-like TAMs primarily secrete pro-inflammatory cytokines like interleukin 12 (IL-12), tumor necrosis factor (TNF)-α, C-X-C motif chemokine 10 (CXCL-10), and interferon (IFN)-γ. Additionally, they produce high levels of nitric oxide synthase, which has an anti-tumor effect (34, 35).

TAMs are macrophages that have developed M2-like behavior, according to the current consensus among experts (36–39). In summary, carcinogenesis and tumor development are significantly influenced by the tumor microenvironment. Since macrophages comprise the majority of the tumor microenvironment, by comprehending their polarization, we may take measures to manage the environment and prevent the tumor from spreading.

### 3.1 Molecular mechanism of polarization of TAMs from M1-type to M2-type

Colony-stimulating factor 1 (CSF-1) and C-C motif ligand 2 are the two most well-documented macrophage recruitment factors and M2 stimulating factors in the literature at this time (CCL2).Macrophage polarization is greatly influenced by CSF-1, which is also overexpressed in the infiltrative margins of a variety of malignancies and is linked to a much higher risk of metastasis (34). Abraham D. et al. observed that when CSF-1 expression was restored in mice with CSF-1 deletion mutations, tumor development and metastasis were accelerated (40). Furthermore, CSF-1 depletion results in a large reduction in macrophage density, which delays tumor progression and significantly inhibits metastasis, as demonstrated by tumor transplantation models (34, 40–42). CCL2 polarizes macrophages toward the pre-tumor phenotype by binding to the surface of macrophages through the C-C chemokine receptor 2 (CCR2) (43). Research has demonstrated that preventing the CCL2-CCR2 connection through gene ablation or antibody treatment greatly reduces the expression of pre-tumor cytokines, delays the onset of metastasis, and increases the longevity of hormonal animals (43–45). Furthermore, a wealth of clinicopathological information supports the correlation between elevated tumor CCl2 concentrations and heightened TAM infiltration as well as metastases (34, 44, 46).

A pro-tumorigenic effector with pro-angiogenic qualities, vascular endothelial growth factor A (VEGF-A) (47–49) stimulates the formation of malignant tumors by causing TAM infiltration and M2 polarization in the presence of IL-4 and IL-10 (50). Gain-of-function studies using xenograft models of skin cancer revealed that VEGF-A overexpression reduced the depletion of macrophages produced by sodium chlorophosphate, hence shortening the survival of the xenografts (50–52). Furthermore, carcinogenesis, development, and metastasis are frequently associated with hyperactivation caused by overexpression or mutation of the epidermal growth factor receptor (EGFR) signaling pathway (53–55). Indeed, EGFR signaling modulates M2-like polarization and macrophage recruitment, which in turn controls TME production. It also directly stimulates the proliferation and invasion of tumor cells (56–58). In a study by Zhang et al. it was shown that inhibition of EGFR signaling in colon cancer cells to regulate cytokine secretion and block M1 to M2 macrophage polarization inhibited tumor cell growth (59).

Apart from the components mentioned above that have been thoroughly examined, there are other newly discovered unique elements that are also involved in the recruitment and polarization of TAMs. For instance, prostaglandin E2 (PGE2) and CSF-1 work together to trans-activate the CSF-1 receptor (CSF-1R), which promotes M2 polarization. PGE2-induced macrophage infiltration was significantly inhibited in the absence of CSF-1R (60). Through the down-regulation of phosphatase and tensin homologue (PTEN) expression and activation of the PI3K/AKT signaling pathway to induce M2 macrophage polarization, Zhao et al. showed that exosome miR-934 derived from colorectal cancer cells promotes M2 polarization (61), which in turn promotes tumor progression.IL-4 and IL-13 secreted by Helper T cell 2 (TH2) aid in the polarization of the M2 phenotype (62). By depleting arginine, IL-4 and IL-13 restrict the generation of nitric oxide, an inflammatory mediator that leads to the loss of the M1 phenotype and encourages polarization towards the M2 phenotype through the cytokines IL-4 and IL-13 (63). Additionally, glutamine ligase (GLUL), which catalyzes the conversion of glutamate to glutamine, can encourage TAM polarization toward the M2 phenotype (64). In turn, it exerts a pro-tumorigenic effect.

Notably, hypoxia is a major promoter of macrophage recruitment and polarization in TME, and it is present in the majority of solid tumors. Hypoxia-induced chemokines such as CCL2, C-C motif ligand 5 (CCL5), CSF-1, VEGF, Semaphorin 3A (Sema3A), endothelial cell monocyte-activating polypeptide II (EMAP-II), endothelin, stromal cell-derived factor 1α (SDF1α), eosinophilic cytokines Eotaxin and Oncostatin M, and others are crucial for TAMS migration to hypoxic regions (39, 65). High mobility group protein B1 (HMGB1) is most typically linked to hypoxia-induced macrophage polarization.HMGB1 is overexpressed in numerous solid tumors and has been linked to skin carcinogenesis (66), inflammatory responses in hepatocellular carcinoma (67), and colon cancer (68). Additionally, Huber et al.’s study revealed that patients with metastatic melanoma had serum levels of HMGB1 that were higher than those of healthy people. They also used flow cytometry to analyze isolated tumors and discovered that the expression and release of HMGB1 within the tumors promoted the accumulation of M2-like macrophages in the microenvironment (69).

In summary, the regulation of TAMs polarization is crucial in relation to tumor development, and TAMs are the main cells involved in efferocytosis (1, 2). Therefore, we can control or delay tumor development by regulating the polarization of TAMs and thus efferocytosis.

## 4 Efferocytosis in the tumor microenvironment

During the growth of tumors, cell death (apoptosis or necrosis) is a regular occurrence. The evacuation of dead cell corpses from the tumor microenvironment (TME) through efferocytosis is a common immunosuppressive phenomena (70), with significant implications on the immunological phenotype within the TME. Growth arrest-specific protein 6 (GAS6) and protein S (PROS1) function as bridging ligands when the TAM (TYRO3, AXL, MERTK) receptor interacts to phosphatidylserine (PS) on apoptotic cells in TAMs, causing efferocytosis (71).

For macrophages and epithelial cells to be effectively efferocytosed, MERTK must be increased during postnatal remodeling (72). Jamie et al.’s study (73) looked at the impact of postnatal mammary gland remodeling and resurfacing on the development and spread of spontaneous breast cancers. Using immunocompetent mice, they found that during postnatal resurfacing, an increase in M2-like macrophages, wound repair, and an amplification of immune-suppressing cytokines were induced by the stromal response to significant tumor cell death. This ultimately resulted in a 10-fold increase in metastasis. In the meantime, MERTK controlled the stroma’s microenvironmental alterations. Their research revealed the vital roles that efferocytosis and MERTK play in the process of mesenchymal wound healing and remodeling, as well as the significance of efferocytosis in the tumor microenvironment. proving once more how strongly efferocytosis and cancer are related. Bondanza et al. (74) discovered that inhibiting efferocytosis by disrupting PS-phagocytic connections via Annexin V effectively reduced tumor development and metastasis. Annexin V has a strong affinity for PS on the surfaces of apoptotic and necrotic cells, which influences macrophage uptake. Stach et al. (75) have also demonstrated this process. Thus, we can intervene in tumor development and spread by inhibiting PS-dependent recognition and immunosuppressive clearance of necrotic cells, also known as efferocytosis.

Werfel et al.’s work (76) showed that efferocytosis and indoleamine-2,3-dioxygenase (IDO1, an immunomodulatory factor known to enhance maternal-fetal antigen tolerance, respectively) are two ways in which apoptotic and necrotic tumor cells promote tumor growth. Apoptotic or necrotic tumor cells are eliminated by efferocytosis action in a Her2-positive breast cancer model, while immunosuppressive cytokines, myeloid-derived suppressor cells (MDSCs), and regulatory T lymphocytes (Tregs) proliferate. Secondary necrosis of apoptotic cells was induced if the efferocytosis effect was stopped, however this did not stop the rise in immunosuppressive cytokines, MDSC, and Treg. It was discovered that cytarabine increased type II interferon (IFN-γ) expression, which in turn increased IDO1 expression. The immunosuppressive phenotype brought on by necrotic and apoptotic cells was also inhibited and tumor metastasis was prevented when cytarabine and IDO1 expression were inhibited together. This further illustrated the tight connection between tumor development and the burying action of efferocytosis.

M2-type macrophages were detected in prostate cancer (PCa) bone tumors in mice during an investigation by Jones et al. (77) into the mechanism of PCa skeletal metastasis. Compared to M1-type macrophages, *in vitro*-induced M2-type macrophages were superior at phagocytosing (efferocytosing) apoptotic tumor cells. Furthermore, it has been demonstrated in experiments that the mere existence of macrophages is not enough to trigger the formation of tumors, indicating the significance of efferocytosis function. A bridging protein called milk fat globule epidermal growth factor 8 (MFG-E8) has been linked to the suppression of pro-inflammatory reactions and the promotion of efferocytosis. The enhancement of M2 polarization by MFG-E8-mediated efferocytosis was examined by Soki et al. (78). It was discovered that the STAT3/SOCS3 pathway caused PCa-associated macrophages to become polarized through MFG-E8-mediated efferocytosis. PCa progression was caused by impaired macrophage efferocytosis function in mice. When PCa is paired with bone metastases, proinflammatory cytokines, particularly CXCL5, are expressed when PCa cells are efferocytosed *in vitro* by macrophages, allowing the tumor cells to proliferate and thrive in an inflammatory milieu. According to these research, macrophages polarize into tumor-promoting M2 cells through a special mechanism called efferocytosis, which is facilitated by MFG-E8.Roca et al. (79) have provided evidence for this mechanism, reporting higher serum levels of CXCL5, a chemokine with a C-X-C motif, and increased efferocytosis activity in PCa bone metastases. Studies aimed at preventing tumor progression can focus on efferocytosis inhibition or disruption, as this process expedites the growth of tumors.

The E3 ubiquitin ligase (SIAH2)-nuclear respiratory factor 1 (NRF1) axis affects tumor mitochondrial activity, TAMs polarization, and cell death. It also supports tumor maintenance by altering the TME, as demonstrated by Ma et al’s (80) study on breast cancer. Their research also revealed that tumor cells experienced secondary necrosis due to inhibited macrophage polarization and were more vulnerable to apoptosis when efferocytosis was hampered by hypoxic inhibition of NRF1 degradation. Furthermore, cytoburial-induced cytokines linked to wound healing, such as IL-4, IL-10, IL-13, and transforming growth factor B (TGF-B), cause widespread cell necrosis in TME, which furthers the progression of metastatic tumors (73).

## 5 Conclusion

The current understanding of the mechanism of action between efferocytosis and malignancies is limited to the impact of macrophage polarization on the tumor microenvironment and has not been fully investigated. The mechanism of efferocytosis-tumor interactions cannot be disregarded because tumor cells and their microenvironment interact to cause tumor heterogeneity and to promote tumor development. Furthermore, macrophages are involved in many different aspects of the microenvironment that supports the formation and development of tumors. Research on the connection between efferocytosis and tumor progression has produced positive results recently, particularly in the cases of breast cancer, prostate cancer, and hematological system tumors. It has also been discovered that there is a close relationship between efferocytosis and tumor progression. This is due to advancements in science, technology, and medical technology. Unfortunately, there are still a lot of gaps in our understanding of the relationship between tumors and efferocytosis action. One such gap is the lack of thorough research in this area, particularly with regard to lung cancer, which is the second most common cause of cancer incidence and the primary cause of cancer deaths (81), as well as the malignant tumor with the highest incidence and mortality rates in our nation (82), Since the low prevalence of early lung cancer screening makes it extremely ground-breaking research to identify pertinent pathways that can impede the disease’s progression. We are not at a loss for diseases that afflict the entire human race thanks to the role of efferocytosis, which offers fresh perspectives and productive avenues for the research of tumor progression.

## Author contributions

YX: Writing – original draft, Writing – review & editing, Conceptualization, Data curation, Investigation. ZL: Writing – review & editing. SS: Conceptualization, Writing – review & editing. FA: Supervision, Writing – review & editing. GY: Funding acquisition, Supervision, Writing – review & editing.
